# Is Embryo Cryopreservation Causing Macrosomia—and What Else?

**DOI:** 10.3389/fendo.2020.00019

**Published:** 2020-01-28

**Authors:** Raoul Orvieto, Michal Kirshenbaum, Norbert Gleicher

**Affiliations:** ^1^Department of Obstetrics and Gynecology, Chaim Sheba Medical Center, Ramat Gan, Israel; ^2^The Tarnesby-Tarnowski Chair for Family Planning and Fertility Regulation, Sackler Faculty of Medicine, Tel-Aviv University, Tel Aviv, Israel; ^3^The Center for Human Reproduction, New York, NY, United States; ^4^The Foundation for Reproductive Medicine, New York, NY, United States; ^5^Stem Cell Biology and Molecular Embryology Laboratory, Rockefeller University, New York, NY, United States

**Keywords:** cryopreservation, frozen-thawed embryo transfer, IVF, pregnancy complications, macrosomia

## Abstract

The number of embryos transferred during an IVF cycle is directly related to the high incidence of multiple births, which is the culprit of perinatal morbidity. Therefore, single fresh embryo transfer (ET) strategy, or freeze-all, followed by a single frozen-thawed embryo transfer (FET) cycle, may dramatically reduce the rate of multiple births, without compromising the cumulative live birth rates (LBRs). A literature review was conducted for all available evidences assessing obstetrics and perinatal outcomes associated with FET compared to fresh ET and natural conception. While studies comparing fresh and FET cycles in normal responders have yielded conflicting results for pregnancy rate, FET was associated with lower risk of prematurity and low birth weight and increased risk of large for gestational age (LGA) and/or macrosomic in singletons, when compared with fresh ET. Macrosomic/LGA births have a higher risk of fetal hypoxia, stillbirth, shoulder dystocia, perineal lacerations, cesarean section, postpartum hemorrhage and neonatal metabolic disturbances at birth. Nonetheless, it seems that other than higher risk of fetal macrosomia, there are additional obstetric complications associated with FET. The relative risk of hypertensive disorders in pregnancy, as well as perinatal mortality were also demonstrated to be increased in FET compared with singletons from fresh ET and natural conception. Therefore, when considering elective freeze-all policy, in addition to LBR and the risk of ovarian hyperstimulation syndrome, physicians should consider the aforementioned increased FET cycles' pregnancy complications, including LGA/ macrosomia, hypertensive disorders of pregnancy, as well as perinatal mortality.

## Background

The number of embryos transferred during an IVF cycle is directly related to the high incidence of multiple births, which are the culprit of perinatal morbidity. Therefore, the single fresh embryo transfer (ET) strategy, or freeze-all, followed by a single frozen-thawed embryo transfer (FET) cycle, may dramatically reduce the rate of multiple births, without compromising the cumulative live birth rates (LBRs) (1). This trend toward single ET results in the cryopreservation of the surplus embryos for future replacement.

However, when considering elective freeze-all policy, in addition to LBR and the risk of ovarian hyperstimulation syndrome (OHSS), physicians should consider a variety of pregnancy complications that might be associated with FET cycles. In the present review we aim to present and discuss pregnancy complications of patients undergoing FET cycles. This will aid both fertility specialists' counseling and their patients in adjusting the appropriate treatment strategy when considering the freeze-all policy.

## Live Birth Rates: Fresh vs. FET Cycles

Several studies comparing FET and fresh cycles in normal responders have demonstrated a significantly higher clinical pregnancy rate per transfer in the FET vs. the fresh cycles (2, 3). According to Shapiro et al. (2), the advantages of FET over fresh cycles might be due to the adverse effect of controlled ovarian hyperstimulation (COH) on endometrial receptivity, leading to advanced receptive phase, which interferes with embryo–endometrium synchronization.

On the other hand, Wong et al. (4), while evaluating the effectiveness and safety of the freeze-all compared to the conventional IVF/ICSI strategies in patients undergoing assisted reproductive technology (ART) revealed moderate-quality evidence, showing that one strategy is not superior to the other in terms of cumulative LBRs. Moreover, low-quality evidence suggests that not performing a fresh ET lowers the OHSS risk for women at risk of OHSS.

In addition, Bosdou et al. (5) conducted a meta-analysis that consisted of 4 RCTs (*n* = 3,255 patients), comparing the first frozen ET (in a freeze-only cycle strategy) to a fresh ET in normal responders and 4 RCTs (*n* = 2,010 patients) that conducted the same comparison in high responders. In high responders, a significantly higher probability of live birth was observed in the FET group when compared with the fresh ET group, while the probability of live birth was not significantly different between the FET group and the fresh ET group in normal responders. Moreover, in agreement with Bosdou et al. (5), Roque et al. (6) also observed a significant higher LBRs by elective FET than by fresh ET in hyper-responders, but not in normo-responders. However, they could not observe any significant difference in cumulative LBR in the overall population.

Two recent large prospectively randomized multicenter studies of Chinese Han populations offered further evidence that the effects of all-freeze cycles vary with patient populations. Both studies were practically identical in design in that they tested all-freeze cycle outcomes with eSETs in comparison to fresh transfers. There was only one difference; Wei et al. transferred single embryos at blastocyst stage (7), while Shi et al. transferred at cleavage stage (8). With blastocyst-stage transfer all-freeze cycles demonstrated significantly improved IVF outcomes, while with cleavage stage transfer, they did not. The only possible explanation for these discrepant findings is that patients with blastocyst-stage transfers are favorably selected (i.e., smilar to high-responders), while patients with cleavage-stage embryo transfer are much less favorably selected.

To summarize, elective FET might have an advantage in first ETs over fresh ET in good prognosis, but not in average and certainly not in poor prognosis patients, with no difference in cumulative LBRs.

## Comparison of Art Pregnancies vs. Naturally Concieved Pregnancies

A systematic review by Pandey et al. (9) of 30 cohort studies has demonstrated that ART singleton pregnancies were associated with an increased risk of adverse pregnancy outcomes and maternal complications. IVF/ICSI singleton pregnancies were associated with higher risks of ante-partum hemorrhage, hypertensive disorders of pregnancy, gestational diabetes, preterm rupture of membranes, preterm birth (PTB), induction of labor and small for gestational age (SGA), Cesarean section, low birthweight (LBW), congenital anomalies and perinatal mortality. Another retrospective analysis evaluating 123,383 diverse live births has concluded that SGA was associated with increased mortality, while large for gestational age (LGA) and appropriate-for-gestational age (AGA) had similar likelihoods of death (10).

Luke et al. evaluate the risk of severe maternal morbidity by maternal fertility status and for IVF pregnancies, by oocyte source. Compared to fertile women, subfertile, and IVF-treated women had increased risks for blood transfusion and third- or fourth-degree perineal laceration. Also, compared to fertile women, the risk of unplanned hysterectomy and ruptured uterus was increased for IVF-treated women (11).

Qin et al. (12) have studied adverse pregnancy outcomes and pregnancy-related complications in singleton pregnancies after ART, compared with natural conception (NC). Their meta-analysis consisting of 50 cohort studies comprising 161,370 ART and 2,280,241 spontaneously conceived singleton pregnancies demonstrated significantly increased risk of pregnancy-induced hypertension (30%), gestational diabetes mellitus (31%), placenta previa (271%), placental abruption (83%), antepartum hemorrhage (111%), postpartum hemorrhage (29%), polyhydramnios (74%), oligohydramnios (114%), cesarean sections (58%), PTB (71%), very PTB (112%), LBW (61%), very LBW (112%), SGA (35%), perinatal mortality (61%) and congenital malformation (37%). They therefore suggested that ART singleton pregnancies should be considered at high risk for obstetric complications and treated accordingly.

The underlying mechanisms related to the association between ART and adverse outcomes in the singleton pregnancies are unknown. One possible explanation is that the ART procedures, or the causes of infertility, or a combination of these might be responsible for the observed increased risks of adverse ART pregnancies outcomes. According to a study by Vidal et al. (13), an increased risk of LBW was observed with fresh cycles compared with FET cycles. Given that FET cycles do not involve COH, they suggested that the association of fresh cycles with low LBW might result from the COH, rather than the cryopreservation process itself. Additional studies have also supported the detrimental effect of COH on placental implantation with the consequent increased incidence of ischemic placental disease (IPD) (14, 15). Imudia et al. studied the obstetric and neonatal outcomes offresh IVF and demonstrated that peak estradiol level surpassing the 90th percentile (12,661 pmol/L in this study) was associated with higher risk of pre-eclampsia and SGA neonates (15).

Several studies have demonstrated that other parameters associated with the ART procedures themselves, such as the culture media, the delayed fertilization of the oocyte, the duration of time in culture, the freezing and thawing procedures, the manipulation of gametes and embryos, the altered hormonal environment at the time of implantation and the medications used to support early pregnancy, or a combination of these, might increase the risk of adverse outcomes (16–19).

On the other hand, other studies could not relate the ART procedures associated with IVF and ICSI to the adverse perinatal outcomes, since subfertile women conceiving without the aid of ART were also demonstrated to exhibit an increased risk of obstetrics complications (20–22). Therefore, the uncertainty regarding the underlying mechanisms involved in increasing obstetric risks following ART warrants further research.

## FET Pregnancies

Several studies suggested that children born following FET have similar or in most areas even better perinatal outcome, as compared to children born after fresh ET (23, 24). Moreover, while FET was shown to be associated with lower risk of prematurity and LBW in singletons, when compared with fresh ET, there is a growing concern that children born after FET have increased risk of LGA and/or macrosomia (25). Macrosomic/LGA births have a higher risk of fetal hypoxia, stillbirth, shoulder dystocia, cesarean section, postpartum hemorrhage, perineal lacerations and neonatal metabolic disorders at birth (26).

Luke et al. (27) found that in siblings sharing the same mother, children born after FET had a higher risk of LGA than their siblings born after fresh ET (27). Berntsen and Pinborg (28) evaluated the association between FET and LGA and/or macrosomia. Their meta-analysis, consisting of 10 studies on LGA and 6 on macrosomia has revealed that the risk of LGA in FET was increased 1.5-fold and 1.3-fold compared to fresh cycles and NC, respectively. Similarly, they observed 1.7-fold and 1.4-fold increased risk of macrosomia in FET compared to fresh ET and NC, respectively. Whether the increased risk of LGA and macrosomia is associated with higher long-term health risks remains uncertain. Of notice, Zhang et al. (29) have studied large number of women with polycystic ovary syndrome, randomized to either conventional embryo transfer or Freeze-all strategy and showed a similar effect of FET on the risk of LGA. Singleton infants born after FET were more likely to be LGA than those born after fresh ET and the incidence of preeclampsia was significantly higher in the FET than in the fresh ET group, while the risks of gestational diabetes mellitus, PTB and SGA were not significantly different between the fresh and frozen ET groups in both singleton and twin births.

In their retrospective cohort study, evaluating the effect of fresh, compared with frozen embryo transfers on neonatal and pediatric weight and weight gain trajectory, Ainsworth et al. (30) have confirmed the association between frozen embryo transfer and increased birth weight, but the association did not persist when controlling for confounding maternal factors. Moreover, they found no effect of fresh vs. frozen embryo transfer on neonatal weight and childhood weight gain trajectory.

The underlying patophysiology of increased risk of LGA and macrosomia in FET singletons remains uncertain. As mentioned earlier, several possible factors may play a role, i.e., the parental characteristics, as well as the freezing-thawing procedures *per se*, which might induce epigenetic changes during early embryonic stages that alter the intrauterine growth potential in FET offspring.

Moreover, since the uterine environment in frozen cycles is generally more “natural” than that following vigorous COH in fresh cycles, probably the synchronization between the embryos and the endometrium at the time of embryos transfer is also more precise following FET than a fresh transfer. These factors can explain findings of PTB and SGA and LBW babies in fresh ET. Nevertheless, an asynchrony between the endometrium and the embryo might also occur in FET cycles with the consequent influence on fetal growth and development that results in increased birth weight.

The preimplantation period is characterized by significant epigenetic changes, consisting of different methylation patterns, processes that are crucial for embryonic differentiation and growth (31). Wang et al. (32) observed significant differences in methylation patterns of genes important for embryonic growth, H19's and IGF2's differentially methylated domains, between mid-gestation mouse fetuses conceived either naturally or following transfer of frozen or fresh IVF embryos into pseudopregnant mice (32). Both IVF groups showed loss of methylation in both genes' differentially methylated domains, although a greater loss was seen in the FET group. This was correlated with aberrant gene expression compared with controls, while to a lesser degree in the frozen group than the fresh. The authors therefore proposed an embryonal compensatory response induced by vitrification, overcoming the detrimental effects of COH on the oocyte, potentially adjusting for a degree of normalization of gene expression. Several other studies have also demonstrated resembling changes in other genes related to growth, with FET embryos revealing gene expression and epigenetic patterns more similar to NC (33, 34). These might suggest a potential mechanism of vitrification, normalizing the effect of COH on the oocyte epigenetics, with consequentially larger newborns.

## FET and Other Pregnancy Complications

In Maheswashwari et al.'s systematic review (35), the authors concluded that the relative risk of hypertensive disorders in pregnancy in the FET group was higher than in the fresh ET group (RR 1.29). Wennerholm et al. (36), studying the CoNARTaS Nordic cohort study, have also demonstrated that perinatal mortality was increased in FET vs. singletons from fresh ET and NC. Placental related complications resulting in higher perinatal mortality, might all be related to alterations in the implantation and early fetal developmental stages following FET. Since IPD is associated with higher risk of pre-eclampsia and SGA, rather than LGA, as observed in FET, it should be further elucidated whether slow freezing or vitrifying, or the different modes of endometrial preparations might modify the risk of IPD and its associated disorders, such as pre-eclampsia, abruption and SGA.

Trying to deal with the aforementioned questions, Johnson et al. (37) compared pregnancies resulting from FET cycles with those resulting from fresh cycle aiming to evaluate their association with the development of IPD. FET cycles demonstrated a lower risk of IPD or intrauterine fetal demise due to placental insufficiency and a lower risk of small for gestational age than fresh ET, with similar findings before and after adoption of vitrification. However, including only singleton pregnancies in the analysis, resulted in loss of a statistically significant difference of the primary outcome between fresh and FETs. Moreover, while pre-eclampsia was more common in FET cycles, this difference was not statistically significant. Excessive peak E2 level was associated with a higher risk of IPD among fresh cycles, although this difference did not achieve statistical significance.

Sha et al. (38) provided an updated comparison of pregnancy-related complications and adverse perinatal outcomes of pregnancies conceived after FET versus fresh ET. Evaluating 31 eligible studies has revealed that pregnancies resulting from FET were associated with lower relative risks of placental abruption, placenta previa, LBW, very LBW, very PTB, SGA age, and perinatal mortality, as compared with fresh ET. Nonetheless, pregnancies occurring from FET were associated with increased risks of pregnancy-induced hypertension, postpartum hemorrhage, and LGA, as compared with fresh ET. There were no between-group differences in the risks of gestational diabetes mellitus, preterm premature rupture of the membranes, and PTB.

Hwang et al. (39) have retrospectively compared neonatal health outcomes after fresh vs. FET. They demonstrated that compared with infants conceived from fresh embryos, those born to mothers who underwent FET were less likely to be SGA and LBW, but more likely to be LGA and to experience greater odds of infectious, respiratory and neurologic abnormalities.

Green et al. evaluated prepubertal children who were conceived following fresh or frozen ET or NC (40). They found that children born following fresh ET had higher levels of insulin like growth factor I (IGF-I) and more favorable lipid profiles compared with those conceived spontaneously. Moreover, children born after FET had higher levels of insulin-like growth factor II (IGF-II) and lower levels of insulin-like growth factor binding protein 3 (IGFBP-3) than children conceived naturally. These findings underline the fact that while FET may result in fewer obstetric complications than fresh ET, these children still demonstrate differences from those conceived spontaneously.

## Type of Endometrial Preparation

Orvieto et al. (41) evaluated the outcome of natural cycle FET with modified luteal support vs. artifical cycle (AC). Implantation, clinical and ongoing pregnancy rates were found to be significantly higher in patients undergoing the natural cycle FET with the modified luteal support as compared to AC FET. Melnick et al. (42) compared pregnancy outcomes between natural cycle and AC in patients undergoing FET of euploid blastocysts. In accordance with the previous study, ongoing pregnancy rate and live birth rate were higher in NC FETs.

Recent study suggested an association between the presence or absence of corpus luteum (CL) in ART cycles and pre-eclampsia (43). Higher rates of pre-eclampsia were observed in AC FET cycles, where no CL is present, compared to natural or stimulated FET cycles where one or more CL occur. Ginström Ernstad et al. (44) have demonstrated that programmed FET was associated with increased risks of hypertensive disorders in pregnancy, post-partum hemorrhage and Cesarean section, both in comparison to natural and stimulated FET. These increased risks were also observed for FET compared to spontaneous conception and fresh IVF. Moreover, singletons born following AC FET had a greater risk of macrosomia and postterm birth compared to singletons born following FET with a presence of a corpus luteum (i/e., stimulated and natural cycles), while no significant differences were observed for LBW and PTB. Accordingly, an increased risk of macrosomia and LGA were observed in FET compared to fresh IVF and NC. There were no significant differences in any outcomes between natural and stimulated cycles. In general, FET compared to fresh cycles or NC showed neonatal and maternal outcomes in agreement with earlier studies. The authors concluded that the aforementioned observations support the hypothesis of an association between absence of corpus luteum in AC and obstetric complications.

Jing el al. (45) compared the risks of adverse pregnancy outcomes in patients and their offspring after FET during an AC vs. natural cycle. AC-FET was found to be associated with an increased risk for hypertension disorder and Cesarean section. In multiples, birth weight (2,550 g in AC-FET vs. 2,600 g in natural cycle), gestational age (36.6 vs. 37.1 weeks) were higher in the natural cycle- group than in the AC-FET group, although there were no differences in these variables among singletons. They therefore concluded that compared with natural cycle, AC-FET seemed to have a negative effect on obstetric outcomes. Saito et al. studied pregnancy outcomes of patients who underwent FET and found that patients who conceived by AC had increased risks of hypertensive disorders of pregnancy and placenta accreta and a reduced risk of gestational diabetes mellitus in comparison to those who conceived by FET during a natural-cycle FET (46).

## Conclusions

Elective FET might increase LBRs compared to fresh ET in hyper-responders, but not in normo-responders, with comparable cumulative LBR in the overall population and lower risk of moderate/severe OHSS. Moreover, FET was associated with lower risk of prematurity and LBW and increased risk of LGA and/or macrosomic in singletons, when compared with fresh ET. The relative risk of hypertensive disorders in pregnancy, as well as perinatal mortality were also demonstrated to be increased in FET compared with singletons from fresh ET and NC. Recent studies have related the aforementioned pregnancy complications to programmed FET rather than those following natural and stimulated cycles, supporting the link between absence of corpus luteum in artificial cycle and adverse maternal outcomes.

When considering elective freeze-all policy, in addition to LBR and the risk of OHSS, physicians should consider the aforementioned increased FET cycles' pregnancy complications (Table 1), including LGA/macrosomia, hypertensive disorders of pregnancy, as well as, perinatal mortality. Moreover, FET following natural or stimulated cycles are adviced aiming to reduce adverse outcomes.

**Table 1:** Pros and cons in fresh and FET cycles.

**Fresh cycle**	**FET cycle**
**Adverse pregnancy outcome**	
SGA	LGA
LBW	Macrosomia
	Pre-term
**Pregnancy-related complications**	
Placenta previa	Hypertensive disorder of pregnancy
Placental abruption	Post-partum hemorrhage
**Neonatal complications**	
	Neonatal infectious/hematologic/neurologic conditions

*LBW, low birth weight; LGA, large for gestational age; SGA, small for gestational age*.

## Author Contributions

The authors were actively involved in conceptualization, writing—original draft preparation, reviewing and editing the final draft.

### Conflict of Interest

The authors declare that the research was conducted in the absence of any commercial or financial relationships that could be construed as a potential conflict of interest.
